# Active Gains in brain Using Exercise During Aging (AGUEDA): protocol for a randomized controlled trial

**DOI:** 10.3389/fnhum.2023.1168549

**Published:** 2023-05-22

**Authors:** Patricio Solis-Urra, Cristina Molina-Hidalgo, Yolanda García-Rivero, Claudia Costa-Rodriguez, Jose Mora-Gonzalez, Beatriz Fernandez-Gamez, Marcos Olvera-Rojas, Andrea Coca-Pulido, Angel Toval, Darío Bellón, Alessandro Sclafani, Isabel Martín-Fuentes, Eva María Triviño-Ibañez, Carlos de Teresa, Haiqing Huang, George Grove, Charles H. Hillman, Arthur F. Kramer, Andrés Catena, Francisco B. Ortega, Manuel Gómez-Río, Kirk I. Erickson, Irene Esteban-Cornejo

**Affiliations:** ^1^Department of Physical Education and Sports, Faculty of Sport Sciences, Sport and Health University Research Institute, University of Granada, Granada, Spain; ^2^Servicio de Medicina Nuclear, Hospital Universitario Virgen de las Nieves, Granada, Spain; ^3^Faculty of Education and Social Sciences, Universidad Andrés Bello, Viña del Mar, Chile; ^4^Department of Psychology, University of Pittsburgh, Pittsburgh, PA, United States; ^5^AdventHealth Research Institute, Neuroscience Institute, Orlando, FL, United States; ^6^ibs.GRANADA Instituto de Investigación Biosanitaria, Granada, Spain; ^7^Dirección de Seleccion y Admisión de Alumnos, Universidad de Playa Ancha, Valparaíso, Chile; ^8^Andalusian Centre of Sports Medicine, Consejería de Turismo y Deporte, Granada, Spain; ^9^Center for Cognitive and Brain Health, Northeastern University, Boston, MA, United States; ^10^Department of Psychology, Northeastern University, Boston, MA, United States; ^11^Department of Physical Therapy, Movement, and Rehabilitation Sciences, Northeastern University, Boston, MA, United States; ^12^Beckman Institute, University of Illinois, Urbana, IL, United States; ^13^School of Psychology, University of Granada, Granada, Spain; ^14^Faculty of Sport and Health Sciences, University of Jyväskylä, Jyväskylä, Finland; ^15^Centro de Investigación Biomédica en Red Fisiopatología de la Obesidad y Nutrición, Instituto de Salud Carlos III, Madrid, Spain

**Keywords:** exercise, executive function, Alzheimer’s disease, amyloid beta, brain

## Abstract

Alzheimer’s disease is currently the leading cause of dementia and one of the most expensive, lethal and severe diseases worldwide. Age-related decline in executive function is widespread and plays a key role in subsequent dementia risk. Physical exercise has been proposed as one of the leading non-pharmaceutical approaches to improve executive function and ameliorate cognitive decline. This single-site, two-arm, single-blinded, randomized controlled trial (RCT) will include 90 cognitively normal older adults, aged 65–80 years old. Participants will be randomized to a 24-week resistance exercise program (3 sessions/week, 60 min/session, *n* = 45), or a wait-list control group (*n* = 45) which will be asked to maintain their usual lifestyle. All study outcomes will be assessed at baseline and at 24-weeks after the exercise program, with a subset of selected outcomes assessed at 12-weeks. The primary outcome will be indicated by the change in an executive function composite score assessed with a comprehensive neuropsychological battery and the National Institutes of Health Toolbox Cognition Battery. Secondary outcomes will include changes in brain structure and function and amyloid deposition, other cognitive outcomes, and changes in molecular biomarkers assessed in blood, saliva, and fecal samples, physical function, muscular strength, body composition, mental health, and psychosocial parameters. We expect that the resistance exercise program will have positive effects on executive function and related brain structure and function, and will help to understand the molecular, structural, functional, and psychosocial mechanisms involved.

## 1. Introduction

Dementia is generally characterized by a progressive degeneration of the brain, followed by deterioration of both cognitive abilities and the capacity to perform activities of daily living (Guha, 2014; Petermann-Rocha et al., 2020). Alzheimer’s disease (AD) is currently the leading cause of dementia, and one of the most expensive, lethal and serious diseases (Scheltens et al., 2021). Interestingly, the development of the neuropathological features of AD begins more than 20 years before the onset of related clinical symptoms (Bateman et al., 2012). One of the main markers of neuropathology that can appear before the onset of clinical symptoms is the pathological accumulation of brain amyloid beta (Aβ), which is present in more than 20% of cognitively normal older adults (Jansen et al., 2022). Evidence of pathological levels of this peptide may precede changes in brain structure and function as well as cognitive decline (Erickson et al., 2019); particularly, executive function, which refers to high-level, goal-directed cognitive processes involved in activities of daily living, together with memory performance, are among the earliest compromised cognitive domains (Kirova et al., 2015; Tideman et al., 2022). However, while memory decline is one of the most typical changes in AD, impaired executive function may precede (Harrington et al., 2013; Ho et al., 2018) and be predictive of additional cognitive decline (Kirova et al., 2015; Kim et al., 2020; Shepherd et al., 2021), development of mild cognitive impairment (MCI) and AD (Gibbons et al., 2012), especially at older ages (Mez et al., 2013; Petermann-Rocha et al., 2020), and ultimately being a potential target of lifestyle interventions due to their potentially non-specific cognitive benefits (Erickson et al., 2022).

Dementia is not a natural or inevitable consequence of aging (Verissimo et al., 2022). The risk of experiencing cognitive decline may be ameliorated by a physically active lifestyle (Livingston et al., 2020; Feter et al., 2021; Wu et al., 2022). Indeed, up to 20% of the risk of developing dementia in the world’s population can be attributed to physical inactivity, and it is estimated that around 10 million new cases might be avoided or delayed worldwide each year if older adults meet recommendations for physical activity (World Health Organization [WHO], 2015). Thus, physical exercise (i.e., structured and programmed subset of physical activity) emerges as a potential non-pharmaceutical preventive and cost-efficient treatment for age-related cognitive decline, having an influence at molecular, structural and functional, and behavioral levels (Chan et al., 2021).

Several systematic reviews and meta-analyses have described the beneficial effect of exercise interventions on cognitive domains in older adults, including executive function (Hindin and Zelinski, 2012; Guiney and Machado, 2013; Miller and Taylor-Piliae, 2014; Ohman et al., 2014; Wayne et al., 2014; Lu et al., 2016; Gomes-Osman et al., 2018; Chen et al., 2020), memory (Lu et al., 2016), language (Lu et al., 2016), and general cognition (Miller and Taylor-Piliae, 2014; Lu et al., 2016; Barha et al., 2017; Gomes-Osman et al., 2018; Northey et al., 2018); however, there are also studies showing a lack of clear benefits (Lautenschlager et al., 2008; van Uffelen et al., 2008; Snowden et al., 2011; Tsai et al., 2015; Hong et al., 2018; Lamb et al., 2018; Vidoni et al., 2021). Previously conducted randomized controlled trials (RCTs) have primarily focused on the effects of aerobic exercise (i.e., walking) on cognition in cognitively normal older adults (Erickson et al., 2011, 2019; Stillman et al., 2020; Mendez Colmenares et al., 2021), and in adults at early stages of AD (Zheng et al., 2016; Stillman et al., 2020). Resistance exercise (i.e., muscular strength training) is another type of exercise with the potential for improving cognition (Yoon et al., 2018). Although this type of exercise is included in the physical activity guidelines (Bull et al., 2020), the effects of resistance exercise on cognition have been less frequently studied compared to aerobic exercise (Yoon et al., 2018). Interestingly, recent reviews indicate that resistance exercise interventions (over other types of exercise such as walking or yoga, among others) might have a substantial effect on cognition (Li et al., 2018; Northey et al., 2018; Gallardo-Gomez et al., 2022), including at relatively low doses (Gallardo-Gomez et al., 2022). In general, previous studies have found a positive effect of resistance exercise on executive function (Liu-Ambrose et al., 2008; Brown et al., 2009; Coetsee and Terblanche, 2017) and general cognition (Li et al., 2018) in older adults, while there are less clear results on prevention of cognitive decline (Colcombe and Kramer, 2003). However, these interventions differed from each other, in terms of duration (6 weeks to 1 year) (Liu-Ambrose et al., 2008, 2010; Brown et al., 2009; Mavros et al., 2017), type of resistance exercise (i.e., free weight exercises, elastic band exercises, dumbbells/barbells exercises), and frequency (i.e., 1–3 sessions/week), making it difficult to determine the specific dose and type of resistance exercise needed to improve cognition (Li et al., 2018). Thus, these somewhat inconsistent findings call for more well-designed RCTs with the capacity to clarify the effects of resistance exercise on cognition in older adults.

Potential mechanisms underlying the beneficial effects of exercise on cognition have been recently investigated. A recent conceptual review of this literature suggests three evidence-based levels of mechanisms demonstrated by exercise-based RCTs: (i) *molecular level*, such as increments in brain derived neurotrophic factor (BDNF) and insulin-like growth factor 1 (IGF-1); (ii) *brain structure and function level*, such as preserving hippocampal volume, changes in cortical and white matter morphology and changes in functional connectivity; and (iii) *behavioral level*, such as improvements in mood and sleep (Stillman et al., 2020). Not surprisingly, evidence and discussion around most of these mechanisms are demonstrated by aerobic exercise RCTs. However, different types of exercise (i.e., aerobic or resistance exercise) may involve distinct pathways at each mechanistic level (Vilela et al., 2017). For example, some animal studies suggest that the improvements in cognition derived from resistance exercise may be related to other central and peripheral inflammatory changes (Vilela et al., 2017), or metabolic profile (Serra et al., 2021), and a few human studies highlight the release of neurochemical markers such as BDNF, IGF-1, and homocysteine (Liu-Ambrose and Donaldson, 2009; Herold et al., 2019). On the other hand, effects of other novel brain-related markers of aerobic exercise such as epigenetic factors (Liang et al., 2021; Urdinguio et al., 2021), Cathepsin B (Pedersen, 2019), vascular endothelium growth factor (VEGF), glycosylphosphatidylinositol-specific phospholipase D1 (GPLD1) (Horowitz et al., 2020), clusterin (Horowitz et al., 2020) and central markers such as cerebral blood flow, are underexplored related to resistance exercise interventions in humans. Thus, examining the mechanisms underlying the effects of resistance exercise on cognition is needed, particularly in cognitively normal older adults. To that end, well-designed resistance exercise RCTs to test its effects on cognition, and the potential mechanisms involved should be implemented. In addition, those studies should adequately report the exercise characteristics (i.e., type, frequency, and volume) to establish coherent and robust evidenced-based exercise recommendations (Huang et al., 2021).

Here, we propose the Active Gains in brain Using Exercise During Aging (AGUEDA) trial to shed light on the research gaps related to resistance exercise. This paper describes the rationale and methodology of the AGUEDA RCT. The primary aim of the study is to investigate the effects of a 24-week resistance exercise program on executive function in cognitively normal older adults. The secondary aims are (i) to examine the effects of resistance exercise on brain structural and functional markers (e.g., gray and white matter measures, functional connectivity or cerebral blood flow), brain Aβ deposition, peripheral molecular markers (e.g., BDNF, IGF-1, and Aβ), and other cognitive outcomes (e.g., memory and language), and (ii) to investigate mediators and moderators of the potential exercise-derived improvements observed in executive function and brain markers. The overall hypothesis is that a 24-week resistance exercise program will improve executive function and related brain health metrics in cognitively normal older adults. In addition, we hypothesized that mechanisms involved in exercise-induced enhancements to executive function are present at molecular, brain structure and function, and behavioral levels.

## 2. Methods and analysis

### 2.1. Design and ethics

**FIGURE 1:**
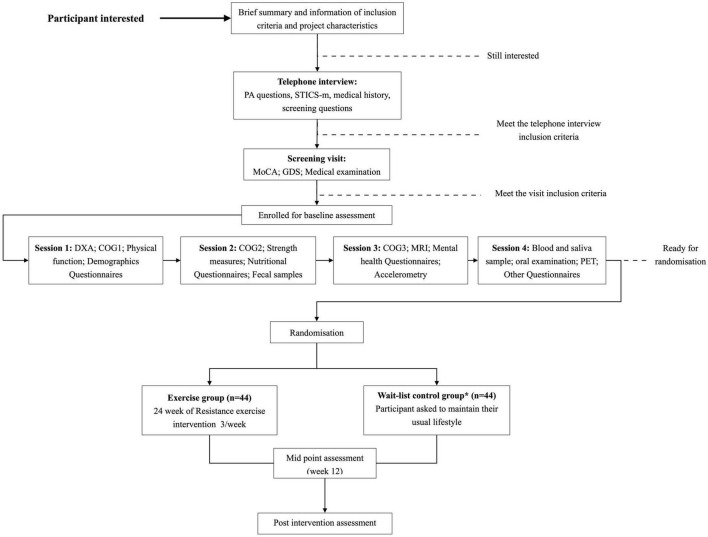
Visual representation of the participant flow in the AGUEDA trial. PA, physical activity; STICS-m, Modified Spanish Telephone Interview of Cognitive Status; MoCA, Montreal Cognitive Assessment; GDS, Geriatric Depression Scale; DXA, dual energy X-ray absorptiometry; MMSE, Mini-Mental State Examination; MRI, Magnetic Resonance Imaging; PET, positron emission tomography. *Wait-list control group is asked to maintain its lifestyles during the 24-week and start the exercise program after post-intervention assessment. ^#^Post-intervention assessment includes the same outcomes that baseline assessment.

The AGUEDA trial is a single-center, two-arm, single-blind RCT in which 90 cognitively normal older adults, aged 65–80 years old, will be randomized into a resistance exercise group (*n* = 45 or a wait-list control group (*n* = 45). A visual representation of the participant flow during the study can be seen in Figure 1. The study will take place in Granada (Spain) at the Sport and Health University Research Institute (iMUDS) and Mind, Brain and Behavior Research Centre (CIMCYC) from the University of Granada, and the Virgen de las Nieves University Hospital. The trial protocol is in accordance with the principles of the Declaration of Helsinki and has been approved by the Research Ethics Board of the Andalusian Health Service (CEIM/CEI Provincial de Granada; #2317-N-19) on May 25th, 2020. The trial is registered on Clinicaltrials.gov (ClinicalTrials.gov Identifier: NCT05186090; Submission date: December 22, 2021). All participants will provide written informed consent once all study details have been explained. A model consent form is publicly available in github.^1^ The study has been designed following the Standard Protocol Items for Randomized Interventional Trials (SPIRIT) (Chan et al., 2013), and the SPIRIT checklist filled for the present study can be found in Supplementary Table 1. In the event that any important protocol modifications are adopted, the study team will communicate them to the trial registry and Research Ethics Board according to the SPIRIT recommendations, and posted in github (see text footnote 1). In addition, all participants of the AGUEDA trial are covered by a social responsibility insurance policy provided by the University of Granada, in case ancillary, post-trial care or compensation are needed. Participants will not receive any monetary incentive for participating in the study.

### 2.2. Recruitment

We began recruitment of community-dwelling older adults in Granada (Spain) city and surrounding areas in March 2021. Strategies to achieve the targeted sample size mainly include mass mailings and social media advertisements, word of mouth, with augmentation of the recruitment strategy by using advertisements in newspapers (TV, radio, and internet). We plan to enroll 90 cognitively normal older adults between the ages of 65 and 80 years, that meet the inclusion and exclusion criteria presented in Table 1. Recruitment, enrollment, and randomization occurs on a rolling basis.

**TABLE 1:** Active Gains in brain Using Exercise During Aging (AGUEDA) inclusion and exclusion criteria for selecting participants.

Inclusion criteria	Exclusion criteria
- Men and women 65–80 years old.	- Ambulatory with pain or regular use of an assisted walking device.
- Able to speak and read fluent Spanish.	- Medical contraindication for inclusion in an exercise program.
- Living in community settings during the study.	- Neurological condition (multiple sclerosis, Parkinson’s disease, dementia) or brain injury (traumatic or stroke).
- Reliable means of transportation.	- Current diagnosis and treatment of a DSM V axis I or II disorder including major depression, and seeing a psychologist, therapist, or psychiatrist in the last year.
- Being physically inactive: (i) not participating in any resistance exercise programs in the last 6 months, and (ii) accumulating less than 600 METs/week of moderate-vigorous physical activity.	- History of major psychiatric illness including schizophrenia, general anxiety disorder, or depression (GDS-30 ≥ 15).
- Classified as cognitively healthy according to STICS-m, MoCA, and MMSE.	- Current treatment for congestive heart failure, angina, uncontrolled arrhythmia, deep venous thrombosis or another cardiovascular event.
	- Myocardial infarction, coronary artery bypass grafting, angioplasty or other cardiac condition in the last year.
	- Current or previous treatment for any type of cancer.
	- Type I diabetes or uncontrolled Type II diabetes defined as insulin dependent.
	- Current treatment for alcohol or substance abuse.
	- Presence of metal implants (e.g., pacemaker, stents, and joint replacement) that would be MRI ineligible.
	- Claustrophobia.
	- Color blindness.
	- Diagnosis of COVID-19 with hospitalization in intensive care unit.
	- Any other consideration that interferes with the study aims and could be a risk to the participant, at the discretion of the researcher

DSM, Diagnostic and Statistical Manual of Mental Disorders; GDS, Geriatric Depression Scale; METs, Metabolic Equivalents; MMSE, Mini-Mental State Examination; MoCA, Montreal Cognitive Assessment; STICS-m, Modified Spanish Telephone Interview of Cognitive Status; MRI, Magnetic Resonance Imaging; COVID-19, coronavirus disease-19.

### 2.3. Screening

#### 2.3.1. Telephone screening

Following recruitment strategies and after the participant’s first contact for general information, an initial telephone interview is performed with those individuals who are potentially interested in participating. Questions about structured exercise and physical activity levels, medical history, Magnetic Resonance Imaging (MRI) compatibility and demographic characteristics are provided by a trained evaluator in the interview. In addition, the Spanish version of the modified Telephone Interview for Cognitive Status (STICS-m) is administered (Munoz-Garcia et al., 2019), and only those that score ≥26 points are eligible to participate. After the interview, an initial check of inclusion/exclusion criteria met by the participants in accordance to their medical history, physical activity levels, MRI safety, and cognitive status is performed before the screening visit.

#### 2.3.2. On-site screening

Before the enrollment of participants in baseline assessments, an on-site screening will be performed. To determinate whether participants meet inclusion/exclusion criteria, this screening includes the Montreal Cognitive Assessment (MoCA) test, the Mini-Mental State Examination (MMSE, performed in a different visit than the MoCA), the Geriatric Depression Scale (GDS) (Izal and Montorio, 1993), and a medical examination conducted by a physician to determinate safety participate in resistance exercise program (Table 1). The MoCA and MMSE both have a maximum of 30-points and are used to identify cognitively normal older adults. We used standardized cut-offs according to age (<71 years, ≥24/30, 71–75 ≥ 22/30, >75, 21/30) and adjusted by years of education for a Spanish population for the MoCA (Ojeda et al., 2016), and ≥25/30 for MMSE. The GDS-30 is used to determine whether the participant meets the exclusion criteria for depressive symptoms. Any participant with a GDS ≥ 15 was excluded from further participation (Fernández-San Martín et al., 2002).

### 2.4. Outcome measurements

A summary of the primary, secondary, and additional outcomes and their time-points of collection is shown in Table 2. All outcome-related measures and analyses are performed by staff who were blinded to the intervention assignment. In brief, the primary outcome of the AGUEDA trial is an executive function composite score that includes several tests assessing domains of executive function such as cognitive flexibility, working memory and inhibitory control (Diamond, 2013). Secondary outcomes include additional cognitive measures (e.g., memory, language, and intelligence), as well as brain structure and function, brain Aβ load and selective peripheral biomarkers. Other health-related outcomes will be evaluated to test for mediation and moderation of biological, physiological, mental health and physical function variables on the potential exercise-derived improvements observed in executive function and brain markers. All protocols can be found in its original language (Spanish) in github (see text footnote 1).

**TABLE 2:** Assessments’ organization and distribution across time-points.

	Telephone-screening	On-site screening	Baseline	Mid-point	Post-intervention
Demographics	x	x			
Medical history	x	x			
Modified Spanish Telephone Interview of Cognitive Status (STICS-m)	x				
Physical activity level (self-reported)	x				
Medications		x			
Family history		x			
Montreal cognitive assessment (MoCA)		x			x
Mini-Mental State Examination (MMSE)		x			x
Geriatric Depression Scale (GDS)		x		x	x
Medical examination		x			
**Primary outcome**					
Executive function tests			x	x	x
**Secondary outcomes**					
Positron emission tomography (PET)			x		x
Magnetic Resonance Imaging (MRI)			x		x
Other cognitive tests			x		x
**Other outcomes**					
Muscular strength			x	x	x
Physical function			x	x	x
Physical activity monitoring			x	x	
Body composition and blood pressure			x		x
Biological samples (blood, fecal and saliva)			x		x
Mental health and other questionnaires			x	x	x

#### 2.4.1. Executive function and other cognitive outcomes

Executive function was selected as the primary outcome based on its importance for general cognitive decline and risk for dementia in older adults (Vaughan and Giovanello, 2010). Briefly, the executive function composite score will be computed using scores from both paper-and-pencil tests (Trail making test A and B and Digit symbols substitution test) and computerized-based National Institutes of Health (NIH) Toolbox tests (Dimensional Card Sort Task, List Sort Working Memory, Picture Sequence and Flanker test), and programmed tests in E-prime software (task-switching, spatial working memory and Stroop test). Individual task scores will be converted to standardized z-scores by subtracting individual raw values minus the baseline group mean, and dividing by the baseline group standard deviation. First, we will verify whether the z-score of each task falls under the proposed executive function domains (i.e., cognitive flexibility, working memory and inhibitory control) using confirmatory factor analysis. Then, the z-score of each task will be equally weighted and pooled into its domain, and then averaged them into an executive function composite score. Additional cognitive outcomes will be assessed such as general cognition, language, verbal memory, visuospatial memory, and crystallized and fluid intelligence. Table 3 shows the cognitive tests and their time point of collection, and details the tests incorporated into the executive function composite score. To ensure accurate assessment, all paper-and-pencil tests will be scored by two different trained staff members and in cases of disagreement, a solution will be resolved by consensus. In addition, the order of the tests (paper-and-pencil and computerized-based tests) will be randomized considering the assessed cognitive domain to minimize fatigue from affecting the results.

**TABLE 3:** Cognitive tests included in the AGUEDA trial.

Cognitive test	Session	Format	Domain(s)	Time	Screening	Baseline	Mid-point	Post-intervention	EF score
Spanish telephone interview for cognitive status (Munoz-Garcia et al., 2019)	Telephone	Verbal	GC	10 min	x				
SCD (Jorm et al., 1997)	Telephone	Verbal	SCD	2 min	x			x	
Montreal cognitive assessment (Ojeda et al., 2016)	On-site screening	Verbal-paper-pencil	GC	10 min	x			x	
Mini-Mental State Examination (Blesa et al., 2001)	On-site screening	Verbal-paper-pencil	GC	10 min	x			x	
Rey auditory verbal learning test (Miranda and Valencia, 1997)	1	Verbal	Verbal memory	40 min		x		x	
Trail making test A and B (Reitan, 1958)	1	Paper-pencil	Cognitive flexibility	5 min		x	x	x	x
Digit symbol substitution test (Wechsler, 2008)	1	Paper-pencil	Processing Speed	5 min		x	x	x	x
Rey–Osterrieth complex figure (Rey, 2009)	1	Paper-pencil	Visuospatial memory	20 min		x		x	
Rapid cognitive screening (Malmstrom and Morley, 2013)	1	Verbal-paper-pencil	Cognitive dysfunction	4 min		x	x	x	
Boston Naming Test (Kaplan et al., 2005)	1	Verbal	Language	7 min		x		x	
National Institutes of Health (NIH) toolbox									
Dimensional card sort task (Zelazo et al., 2013)	2	Computerized	EF	5 min		x	x	x	x
List sort working memory (Zelazo et al., 2013)	2	Computerized	Working memory	5 min		x	x	x	x
Picture sequence (Zelazo et al., 2013)	2	Computerized	Working memory	5 min		x	x	x	x
Flanker (Zelazo et al., 2013)	2	Computerized	EF	5 min		x	x	x	x
Wechsler adult intelligence scale IV (Wechsler, 2008)									
Similarities (Wechsler, 2008)	2	Verbal	CI	5 min		x		x	
Vocabulary (Wechsler, 2008)	2	Verbal	CI	10 min		x		x	
Information (Wechsler, 2008)	2	Verbal	CI	5 min		x		x	
Block design (Wechsler, 2008)	2	Verbal	FI	10 min		x		x	
Matrix reasoning (Wechsler, 2008)	2	Verbal	FI	5 min		x		x	
Visual puzzles (Wechsler, 2008)	2	Verbal	FI	5 min		x		x	
Verbal and semantic fluency test (Rosselli et al., 2000)	2	Verbal	Language	4 min		x	x	x	
Spatial working memory (Erickson et al., 2011)	3	Computerized	Working memory	10 min		x	x	x	x
Task-switching	3	Computerized	EF	10 min		x	x	x	x
Stroop task	3	Computerized	EF	10 min		x	x	x	x

SCD, subjective cognitive decline; EF, executive function; CI, crystallized intelligence; FI, fluid intelligence; GC, general cognition.

#### 2.4.2. Brain structure and function

Brain structure and function will be assessed by MRI at the CIMCYC from the University of Granada. Participants will wear comfortable clothes and sign a specific MRI informed consent. As per the informed consent, participants will have to be compatible with MRI, i.e., not have metallic implants that could be dangerous in the MRI environment and report not being claustrophobic. Standard sequences will be collected for a total acquisition time of approximately 60 min. Table 4 shows the image sequences that will be performed for each participant in acquisition order. A Siemens Magnetom PRISMA Fit 3T scanner with a 64-channel head coil will be used and all brain sequences will have the same slice selection. This slice selection from the field map will be copied to each sequence except for the focal hippocampal sequence, which has its own instructions for determining coverage of the anatomy. Notably, a radiologist will review structural sequences to check for any incidental findings. In cases of incidental findings, the radiologist will contact the participant for further examination. To ensure a minimal loss of data, we will perform a visual inspection and a quality control check of the images on a rolling basis. In addition, custom quality control will be performed including sequence accuracy, conversion to brain imaging data structure (BIDS) format, a check of sequence parameters by examining header information, and quantitative control using MRQC software (Esteban et al., 2017). Briefly, data collected will consist of images providing information about brain volume, surface, shapes and thickness, white matter microstructure, resting state functional MRI and task-evoked fMRI, and cerebral blood flow.

**TABLE 4:** Brain Magnetic Resonance Imaging (MRI) sequences and PET/CT acquisition parameters of the AGUEDA trial.

Measurement	Sequence	Parameters	Acquisition time
MRI	T1-weighted MPRAGE structural	Sagittal, 0.8 mm isotropic resolution, TE/TI/TR = 2.31/1060/2400 ms, FOV = 256 mm, 224 slices	6 min 38 s
	High resolution Hippocampus	Resolution: 0.4 × 0.4 × 2 mm, TE/TR = 79/8830 ms, aligned perpendicular to hippocampus	4 min 53 s
	Resting state EPI	Resolution: 2.5 × 2.5 × 2.5 mm, TE/TR = 40/1000 ms, multiband factor = 8 (CMRR EPI sequence), 64 slices, 480 measurements	8 min 12 s
	fMRI n-back task	Resolution: 2.5 × 2.5 × 2.5 mm, TE/TR = 40/2000 ms, multiband factor = 4, 64 slices, 183 measurements	7 min 12 s
	Diffusion weighted acquisition	Resolution: 2 × 2 × 2 mm, TE/TR = 95.6/2800 ms, multiband factor = 4, *b*-values of 1500, 3000 s/mm2, 64 gradient directions	6 min 18 s
	3D T2 TSE FLAIR	Sagittal, 1 mm isotropic resolution, Turbo spin echo, TE/TI/TR = 388, 2200, 6000 ms, non-selective inversion recovery	7 min 12 s
	pCASL TGSE	3D GRASE pCASL sequence, resolution: 3.1 × 3.1 × 2.5 mm, TE/TR = 23.64/4300 ms, 48 slices, Post-label delay 2 s, background suppression, 10 measurements for labeling and control, four segment readout	5 min 48 s
PET/CT- [90 to 110 min after intravenous injection of [18F]Florbetaben -Neuraceq; Piramal Pharma-]	Computed tomography	400 mA, voltage = 120 kV, 16 s duration, delay time = 2 s, slice thickness = 3 mm, 75 slices, correction = Care Dose, reconstruction = J30s homogeneous/iMAR, FOV = 500 mm	5 min
	Positron emission tomography	Matrix size: 440, activity = 300 MBq (8,1 × 10^–3^ Ci), reconstruction = 10 iterations and 5 subfields, zoom = 2, correction: Attenuation/dispersion/TOF.	20 min

CMRR, center for magnetic resonance research; EPI, echo-planar imaging; FLAIR, fluid attenuated inversion recovery; FOV, field of view; MPRAGE, magnetization prepared rapid gradient echo; pCASL, pseudo-continuous arterial spin labeling; TE, echo time; TI, inversion time; TR, repetition time; TSE, turbo spin-echo; TGSE, turbo gradient spin-echo; VIBE, volumetric interpolated breath-hold examination; PET/CT, positron emission tomography combined computed tomography; mA, milliamperes; kV, kilovoltage; iMAR, iterative metal artifact reduction; Bq, becquerel; Ci, curie; TOF, time of flight.

#### 2.4.3. Brain amyloid beta deposition

The brain Aβ deposition will be assessed before and after intervention using a PET scan Biograph-Vision 600 Edge Positron emission tomography/Computed tomography (PET/CT) digital scanner (Siemens, Erlangen, Germany) at the Virgen de las Nieves University Hospital in Granada. The accumulation of Aβ is a hallmark of AD and may begin up to 20 years before the onset of dementia (Bateman et al., 2012). For the AGUEDA trial, the tracer used will be [^18^F]Florbetaben (Neuraceq; Piramal Pharma). [^18^F]Florbetaben will be injected intravenously into each participant by a professional nurse blinded to intervention in accordance with the applicable regulatory guidelines. PET images will be acquired from participants 90 to 110 min after intravenous injection of 300 megabecquerel (MBq) ± 20% (Villemagne et al., 2011; Sabri et al., 2015a) according to a standardized acquisition and image-processing protocol (Barthel et al., 2011; Sabri et al., 2015b; Minoshima et al., 2016). Before the acquisition of PET images, a low-frequency CT scan will be performed and used for attenuation correction of PET images (5 min). PET/CT imaging (see Table 4 for parameter’s details) will be performed by a blinded specialist physician for all study participants. A visual quality control after each scan will be performed to ensure quality of data. In addition, a neurologist will visually review the PET images to check for any suspected findings, and only in case of coupled clinical symptoms, the neurologist will contact the participant for progression monitoring.

#### 2.4.4. Biological samples

The AGUEDA trial will test for several biomarkers using blood, saliva, and fecal samples. Blood samples will be collected by a nurse with participants in fasting conditions (08:00–10:00 a.m.) at the Virgen de las Nieves University Hospital. Briefly, 6 ethylenediaminetetraacetic acid (EDTA) tubes, 4 citrate tubes, 4 serum tubes and one PAXGENE tube will be collected corresponding to a total of approximately 50 ml. Part of the blood samples obtained from each participant will be directly processed at the Hospital (1 EDTA, 1 citrate and 2 serum tubes), and the remaining samples will be stored at −80°C for future studies. Saliva samples will be collected in a falcon tube and a deep oral examination in a subsample will be conducted by an odontologist at the Hospital, recording number of teeth present, clinical attachment loss, and probing depth at a minimum of three sites in at least two teeth per sextant or in all teeth to asses periodontal disease. Fecal samples will be collected in standardized conditions using plastic sterile containers for each participant and then stored at −80°C for future analysis. Table 5 shows sample type, initial targeted analysis and preliminary analysis plan. Despite this, the analyses will be performed with the best method available at the moment of sample processing. Briefly, blood analytes include traditional cardiovascular risk factors, biochemical measurement, inflammatory, brain-peripheral biomarkers, telomere length, and genotyping. In addition, blood and saliva samples will be used to measure amyloid and tau levels. Finally, fecal samples will be used to perform metagenomic analysis of the gut microbiome for each participant.

**TABLE 5:** Biological samples and targeted analysis plan.

Sample	Analysis target	Preliminary analysis plan
**Blood samples**		
Plasma	Aβ (Amyloid beta) peptides Aβ1-42, Aβ1-40; T-tau and P-tau; Neurofilament light chain (NFL); Vascular endothelial growth factor (VEGF); chemokine C-X-C motif ligand 13 (CXCL-13); IgM-1; IL-17; pancreatic polypeptide (PPY); Adiponectin; Brain-derived neurotrophic factor (BDNF); Cathepsin B; vascular cell adhesion molecule 1 (VACM-1); C-reactive protein (CRP); Interleukin-6 (IL-6); Interleukin-17 (IL-17); Tumor necrosis factor (TNF-Alpha); Klotho; GLP-1, Clusterin	Plasma biomarker Aβ (Amyloid beta) peptides Aβ1-42, Aβ1-40; T-tau and P-tau; Neurofilament light chain -NFL- concentrations will be measured using Single molecule array (Simoa) methods on an HD-X instrument and commercial assays from Quanterix. Key inflammatory biomarkers analyzed in plasma will analyzed with automated blood cell counters and quantified by multiple analyte profiling technology (MILLIPLEX^®^ MAP Human High Sensitivity T-Cell Magnetic Bead Panel, EMD Millipore Corporation, Missouri, U.S.A.) Klotho will be determined using a solid-phase sandwich enzyme-linked immunosorbent assay (Demeditec, Kiel, Germany) according to the manufacturer’s protocol.
Peripheral blood mononuclear cells	Telomere length	A commercial DNA isolation kit (Qiagen, Barcelona, Spain) will be used to extract genomic DNA from isolated PBMCs. Relative telomere length will be determined by quantitative real-time polymerase chain reaction (qRT-PCR) using the telomere/single-copy gene ratio (T/S)
Serum	Insulin-like growth factor 1 (IGF-1); Insulin; Glucose; Glycated Hemoglobin (HbA1c) and Serum fatty acid (SFA-1)	IGF-1, insulin, glucose, glycated hemoglobin (HbA1c) and serum fatty acids (SFA-1) will be analyzed using XMap technology (Luminex Corporation, Austin, TX) and human monoclonal antibodies (Milliplex Map Kit; Millipore, Billerica, MA). Total cholesterol, HDL-C, LDL-C, triglycerides and glucose will be assessed using a spectrophotometer. Insulin will be assessed by chemiluminescence immunoassay.
Whole Blood	miRNA; DNA (genotyped)	Blood specimens will be used for genotyping. The genotypes will be determined using TaqMan genotyping assays. A commercial RNA isolation kit (Qiagen, Barcelona, Spain) will be used to extract total RNA from whole blood samples. The selection of specific miRNAs will be based on the existing bibliographic information. In accordance with this, the selected miRNA will be evaluated using Taqman probes.
**Saliva samples**		
Saliva	Aβ peptides Aβ1-42, Aβ1-40; T-tau and P-tau	To be determinate with the best method available at the moment of analysis
**Fecal samples**		
Fecal	Metagenomic Analysis of the Gut Microbiome	To be determinate with the best method available at the moment of analysis

#### 2.4.5. Physical function

All physical function, muscular strength, and anthropometry will take place at the iMUDS by trained staff (see Table 6). Physical function will be assessed using two validated test batteries specifically designed for older adults: The Senior Fitness Test (SFT) (Langhammer and Stanghelle, 2015) and the Short Physical Performance Battery (SPPB) (Guralnik et al., 1994). Briefly, these batteries assess upper and lower body strength, aerobic capacity, walking speed, balance, and flexibility, and while the SFT provides individual information of each component, the SPPB provides a composite score indicating mobility function (Addison et al., 2018). In addition, aerobic capacity will be assessed by the 2-km walking test (Skantz et al., 2020), and gait parameters (e.g., flight times, contact times, and gait variability) by the optical *OptoGait* system (Gomez Bernal et al., 2016).

**TABLE 6:** Physical health parameters.

Parameter	Instrument	Description
Physical function	Senior fitness test (SFT)	Consists of six functional measures of strength in arms and legs, endurance, balance, agility and flexibility. The tests included are, chair stand test, arm curl test, 6-min walk test, 2-min step test, chair-sit-and reach test, and back scratch test.
	Short physical performance battery (SPPB)	Consist of three test to measure functional status. The tests include are, hierarchical assessment of standing balance, usual walking speed over 4 m and five chair sit-to–stand test.
Muscle strength	Hand dynamometer TKK 5101	Each participant is encouraged to perform the maximal isometric force twice with each hand.
	Gymmex Iso-2 dynamometer	Unilateral left and right-side test of upper and lower muscle strength are performed. The subject is instructed to sub maximally flex and extend the knee and elbow 3 times, and then to complete five maximal repetitions.
Gait parameters	OptoGait system	The participant’s is encouraged to walk during 5 min to the fast comfortable walking pace in 9 m round trip, safely and without running. The middle 5 m are recorder for analysis. The goal is to record at least 150 steps for each participant.
Body composition	Anthropometric measurement	Body weight and height. Head, neck, waist, and hip circumferences.
	DXA scan	Total body, left and right hips and lumbar spine.
	Neck Magnetic Resonance Imaging (MRI)	An MRI sequence is performed to determinate the neck fat. The sequence parameters are: Resolution: 2 × 2 × 2 mm, TE1-2/TR = 1.23–2.46/5.21 ms, flip angle 9°, CAIPIRINHA iPAT factor = 4, FOV = 448 mm; Duration: 21 s. The patients are instructed to hold breath during the acquisition.
Blood pressure	Omron M3, Intellisense	Three reading will be collected to determine systolic and diastolic blood pressure.
Physical activity	Actigraph GT3X + accelerometry device	The participant’s is encouraged to wear the device in the non-dominant wrist during 10 days.

TE, echo time; TR, repetition time; FOV, field of view.

#### 2.4.6. Muscular strength

Muscular strength will be measured with the handgrip strength test and the upper and lower body isokinetic machine (Table 6). In the handgrip strength test, each participant will be encouraged to perform the maximal isometric force twice with each hand using a hand dynamometer (TKK 5101 Grip D, Takey, Tokyo Japan), and the maximum value of each hand will be taken and averaged. Upper and lower body isokinetic strength will be measured using a Gymmex Iso-2 dynamometer (EASYTRCH s.r.l., Italy) according to the previous published protocol (Artero et al., 2012).

#### 2.4.7. Physical activity monitoring

Objectively measured physical activity will be completed using the Actigraph GT3X + accelerometry device (Pensacola, FL, USA), a tri-axial accelerometer, with raw data filtering technology and with a dynamic range of ±6 g (Table 6). Each participant will receive an accelerometer and will be required to wear it for 10 days on the non-dominant wrist including periods of sleep, removing it only while bathing or swimming. Along with the device, a diary will be provided in which the participant will record wearing/non-wearing time along with sleep information. Actilife v.6.13.3 will be used to initialize the device with a sampling rate of 100 Hz. To ensure a minimal loss of data, we will perform an automatic check for quality control of the data with the criteria of 3 valid days on week and 1 valid day of weekend on a rolling basis using the GGIR package (Migueles et al., 2019). In the case of non-valid registration, the participant will complete another 10 days of assessment. This measurement will be performed at baseline and again in week 12 of the intervention.

#### 2.4.8. Anthropometric, body composition and blood pressure measurements

For anthropometric and body composition measurements, participants will be asked to arrive at the IMUDs between 08:30 and 10:30 a.m. after overnight fasting. Body weight (kg) will be measured in triplicate with an electronic scale (SECA 861, Hamburg, Germany) with an accuracy of 100 g. Height (cm) will be assessed with a precision stadiometer (SECA 225, Hamburg, Germany) to the nearest 0.1 cm. Head, neck, waist and hip circumference will be measured with an inextensible tape in triplicate and average values will be calculated. Body composition will be measured using dual energy X-ray absorptiometry (DXA, Discovery Densitometer from Hologic). A total body scan as well as specific assessment of both hips and lumbar spine will be performed to determine body composition and bone density parameters. Blood pressure will be measured with patients seated with the left arm resting on a table at heart level and a proper cuff size is fitted. Validated automated blood pressure monitor (Omron M3, Intellisense) will be used to take blood pressure measurements. Three readings will be collected: immediately, following 5 min of rest, and following a subsequent 2 min interval (Table 6). Blood pressure is will be calculated as the average of the last two BP readings (Williams et al., 2018).

#### 2.4.9. Psychosocial, mental health and other questionnaires

A battery of questionnaires will be administered including the following dimensions: mental health, psychological health, functional ability, medical information, lifestyle behaviors, and others (Table 7). Exercise self-efficacy, social provisions and groups, mood and anxiety, personality traits, social networks, pain, fatigue, etc. have all been shown to either influence exercise adherence or cognition and might explain, mediate, or moderate some of the effects of exercise on cognition (Stillman et al., 2020). As such, we carefully selected a battery of tests that would considerably extend the knowledge on how exercise influences cognitive and brain health.

**TABLE 7:** Psychosocial, mental health and other questionnaires.

Questionnaire	Description
**Mental health**	
Geriatric Depression Scale (GDS) (Izal and Montorio, 1993)	Measures the presence of depressive symptoms in older adults (30 items).
Hospital Anxiety and Depression Scale (Zigmond and Snaith, 1983)	Questions about anxiety, feelings of depression and emotional distress in the last week (14 items).
Perceived Stress Scale (PSS) (Remor, 2006)	Questionnaire that measures the degree of stress perceived in relation to certain life events during the last month (14 items).
Rosenberg Self-Esteem Scale (RSE) (Robins et al., 2001)	By assessing positive and negative feelings about the self, this scale measures global self-esteem (10 items).
Satisfaction With Life Scale (SWLS) (Vazquez et al., 2013)	It measures the degree of satisfaction with the conditions of their life on a scale of 1 to 7. It measures overall life satisfaction (5 items).
UCLA Loneliness Scale (UCLA-L) (Russell et al., 1980)	This scale assesses feelings of loneliness and social isolation (10 items).
**Psychological variables**	
Social media use	Measures the frequency and usefulness given to social networks (5 items).
Social Support Questionnaire	It measures the type and quality of social relationships maintained by the subject and the degree of social support received (11 items).
Big Five Inventory (BFI) (Caprara et al., 1993)	It measures the Big five dimensions of personality: Extraversion, agreeableness, conscientiousness, emotional stability, and openness to experience (44 items).
Short Form 36 Health questionnaire (SF-36) (Alonso, 2003)	Questionnaire that measures quality of life according to eight health-related dimensions: physical functioning, physical limitations, pain, behavioral disturbances due to emotional problems, mental capacity, health perception, social functioning and energy/fatigue perception (36 items).
**Functional ability variables**	
Instrumental activities of daily living (Lawton and Brody, 1969)	Questionnaire assessing daily cognitive and self-care activities in terms of routine and ability (26 items).
International Fitness Scale (IFIS) (Merellano-Navarro et al., 2017)	It subjectively assesses fitness levels by stratifying a population into fitness level groups (5 items).
Mobility and agility questionnaire	Questions about current health status and existing limitations for certain activities (8 items).
Questions about energy and frailty	It measures the energy levels perceived by the subject during the last time, frailty and body weight variation throughout life (9 items)
McGill Pain Questionnaire (Melzack, 1987)	Questionnaire assessing qualitative experiences of pain through affective, sensory, and perceived pain intensity descriptors (16 items).
**Medical history**	
Health history	Details health history, illnesses, injuries, operations and any other related events that have occurred at any time in life.
Medication list	Information on current and permanent prescription and over-the-counter medications, as well as information on visual impairments.
Oral health questionnaire (Schroder et al., 2011)	Questions about the subject’s oral health (10 items)
COVID questionnaire	Questions about COVID-19 information and COVID vaccine status. (40 items)
Adverse event report	Questions about adverse event during the period in the trial participation. (40 items)
**Lifestyle behaviors**	
Drinks questionnaire	Questions about the frequency of intake of coffee and alcoholic beverages (14 items).
Food-frequency Questionnaire (FFQ) (Boylan et al., 2009)	Subjects are asked to indicate the frequency and usual amount of food and beverage consumption using response categories (148 items).
MEDAS-14 (Schroder et al., 2011)	Questionnaire of adherence to the Mediterranean diet (14 items).
Smoking questions (Schroder et al., 2011)	Questions on smoking habits throughout life (4 items).
Sitting time questionnaire	Subjects respond how much time they spend sitting at work, at home, while watching TV or in their free time, and driving on a typical day during weekday and weekends. (8 items)
Physical activity assessment	Questions on the perception and performance of physical activity during life (77 items).
Pittsburgh Sleep Quality Index (PSQI) (Buysse et al., 1989)	Survey that measures the sleep quality (21 items).
**Others**	
Demographic questions (Schroder et al., 2011)	Questions about the family’s economic situation, years of education, and doctor visits (16 items).
Edinburgh Handedness Inventory (EHI) (Oldfield, 1971)	Questions about how often the subject uses one or the other hand for different tasks (9 items).

### 2.5. Randomization

Randomization will occur on a rolling basis and only after the completion of all baseline assessment sessions by the participant to reduce the risk of bias during the assessment. We will use a computerized randomization protocol through the REDCap Software (Harris et al., 2019) that will incorporate a checklist to make sure all assessments have been completed and the data have been adequately entered into the database. We will use a minimization algorithm with equal allocation (1:1) to one of the two study groups: (1) a 24-week resistance exercise program (3 sessions/week, 60 min/session) group, or (2) wait-list control group which will be asked to maintain their usual lifestyle. The wait-list strategy implies that the control group will also receive the exercise program after all the post-intervention assessments of the AGUEDA trial have been completed. To improve the representativeness of the study sample and the generalizability of future findings, stratification by two factors will be considered: age at study entry (≤72, >72 years) and sex (male, female). This procedure will be carried out by a blinded researcher completely external to the project (VCS). After the randomization procedure, the sport scientist in charge of the exercise program will be responsible for communicating with the participant about the group allocation.

### 2.6. Blinding

Due to the nature of the intervention (physical exercise), it is not possible to “blind” the participants, as they will inevitably know their group allocation. However, the principal investigator (PI) and research staff (who do not conduct the exercise sessions) will be blinded to group assignment. Only in the case of an unforeseen event occurring with a participant, the PI will be able to break this blinding condition.

### 2.7. Resistance exercise program

Detailed description of the exercise program is fundamental to ensure that the program can be disseminated and carried out as clearly and similarly as possible by others, and therefore be replicated in any similar context. Therefore, a detailed and broader description of the program will be further extended in the Consensus on Exercise Reporting Template (CERT) study protocol of the AGUEDA trial (in preparation). Briefly, participants assigned to the 24-week resistance exercise program will be required to attend 3 sessions per week, for approximately 60 min each day, at the Sport Center located at the iMUDS. The intervention will consist of a combination of upper and lower body exercises using elastic bands with different resistance levels and the participants’ body weight as the primary resistance. Considering that the participants will be inactive at baseline, we will dedicate the first two weeks of the program to perform exercises for neuromuscular adaptations of both upper and lower body, and for familiarization with the movement patterns and equipment. The exercises will be based on basic movement patterns involving large muscle groups: horizontal traction, vertical traction, horizontal thrust, vertical thrust, hip extension and flexion, hip dominants, knee dominants, anti-rotation, anti-extension, anti-flexion, anti-lateral flexion (Ribeiro et al., 2020). The exercise program is organized into three levels of difficulty, allowing participants to progressively increase the effort intensity while learning and improving their physical performance. In addition, three model sessions have been designed for each of the program levels, and each 60-min session (regardless of the model) will follow the same organization: 1st Warm-up phase (10 min), 2nd Main phase (45 min), and 3rd Cool-down phase (5 min). It is important to note that the maximum training ratio is 1/6, i.e., there can be a maximum of 6 participants per trainer in each session. Load and intensity of the exercises will be defined by: (i) number of repetitions for each set; (ii) elastic band resistance in ascending order with seven intensities (i.e., yellow to gold (Uchida et al., 2016); (iii) difficulty of exercises (i.e., 3 levels); and (iv) time of performance following the “as fast as possible” indication for every repetition.

The prescribed intensity will be reached at 70–80% of the Rating of Perceived Exertion (RPE) (Colado et al., 2014). The RPE is a scale from 1 to 10 (i.e., from very mild to extremely maximal effort) by which participants will be able to indicate their own perception of the effort made during each exercise. This scale will be visible to participants throughout the session. In addition, the intensity will increase progressively, and the adjustment of each participant’s working loads will be performed using a target RPE, modifying execution time and cadence, and the elastic band type. Heart rate will be monitored during all sessions through a Polar H10 monitor with mobile phone applications. Data will be logged second-by-second into the Polar Beat app and Elite HRV which will be linked to the sensor band. In addition, sleep quality and pre- and post- affective response will be recorded in each session (Hardy and Rejeski, 1989). The exercise program will be conducted by a sport science professional with a bachelor degree in Physical Activity and Sports Sciences or master degree in Personal Training. Adherence to the resistance exercise program protocol will be evaluated as session attendance at the program, and a minimum of 80% attendance to all exercise sessions will be required to meet the protocol. To facilitate adherence, when any participant misses an exercise session for any reason, they will have the possibility to attend another exercise group to make up the missed session. Reasons for non-attendance and details about each recovered session will be documented. The sport science professional in charge of the program will encourage the participant to attend all exercise sessions and will be responsible for rescheduling in case of necessity. The control group will be asked to maintain its usual lifestyle and will receive the same resistance exercise program after the post-intervention assessments at week 24. In order to favor the adherence and the interaction with the control group, bimonthly activities will be performed consisting of meetings in which participants play games, engage in relaxing activities, among others. Adherence strategies will be implemented during the intervention program to minimize potential losses, including birthday messages, games-based and social meetings, among others. In both groups, participants will continue with concomitant care and activities. Participants will be invited to the testing at post-intervention irrespectively of their compliance to the exercise intervention protocol.

### 2.8. Adverse events and participant safety

Because the effects of resistance exercise are well tolerated, minor unrelated non-serious adverse events (e.g., allergies, upset stomach) and minor adverse events related to the practice of physical exercise (e.g., myalgia, sweating, and dyspnea) will not be recorded, unless it requires a change of the intervention (e.g., change in a specific exercise due to articular pain). Any situation that may occur, such as injury, emergency or scheduled surgery, will be reported and evaluated by the research team and the PI will be unblinded when necessary. The adverse event will be recorded in a custom form with detailed information of its seriousness, severity, chronicity, and resolution. Thus, adverse events as well as any possible effects produced by the assessments or the exercise intervention will be carefully monitored, recorded and reported in REDCap. The severity of the adverse event will be determined using common terminology criteria for adverse events (i.e., mild, moderate, severe) (Freites-Martinez et al., 2021), and any adverse event rated category three will be considered as a serious adverse event. If the situation is considered unsafe to continue in the study, temporary or definitive exclusion from the project will be discussed and reported. In the case of temporary exclusion (e.g., any joint lesion) the last exercise session will be recorded, and a physician clearance will be required to rejoin to resistance exercise program.

### 2.9. Data management and dissemination policy

The AGUEDA trial will use two data storage systems: (i) the REDCap platform is an online platform designed to store and manage electronic data and create research databases for clinical trials and translational research. The AGUEDA trial will use this platform for the storage and management of all non-imaging data (e.g., behavioral or cognitive data). This platform will also contain the data entry forms that can be downloaded and printed if necessary. The database of the project will be generated directly from REDCap; (ii) the AGUEDA desktop computer will also contain all participants’ data collected on paper-based forms, that will be scanned and stored, and the MRI and PET image data. It is important to note that storing all the data in REDCap ensures that all the information is safe in case the server is damaged for any reason (Harris et al., 2019). In addition, the database will be automatically stored once a week on a remote hard drive with the data collected and registered at the moment of storing. All physical copies of data collection forms and documents will be stored in a locked cabinet also located at the iMUDS. From the moment participants are enrolled in the trial, we will assign them a code that will be used in all forms and data collection reports, ensuring anonymity of all data. The PI will have continuous access to the final dataset. Participants authorize in the informed consent that their pseudonymized data may be made available for secondary research importantly, once the participant is enrolled and before randomization, all data will be counted, checked for quality control purposes, and archived following the storage protocol described above by the research team, performing these same steps at the three measurement points. Specifically, quality control includes visual immediate inspection of imaging data (MRI and PET/CT), double correction of paper-pencil cognitive test and check limit of real data (ranges and valid type and values) in REDCap.

Data access will operate under the findability, accessibility, interoperability, and reusability (FAIR) principles, and code of analysis will be made publicly available. We will consider governance, ethical and shared trial oversight, and expectations that trials will adhere to the best practice of the day. The results of this study, whether positive, negative, or inconclusive, will be published in peer-reviewed journals as well as at national and international conferences. The study findings will be also released to the participants, physicians, and the general medical community through social media and press. Authorship eligibility will be handled by the PI according to the International Committee of Medical Journal Editors and all authors will contribute to the final revision of each manuscript.

### 2.10. Power and analytic strategy

To calculate the sample size of the AGUEDA trial, we based our estimates on the AGUEDA primary outcome, i.e., change on the executive function composite score over a 24-weeks resistance exercise period. To calculate the sample size, we based the power analysis on a meta-analysis which showed that the effect size of exercise intervention on executive function in older adults was 0.34 (95% Confidence interval = 0.22 to 0.47) (Northey et al., 2018), with a two-tailed alpha at 0.05 and a power of 80%. Thus, we need a sample size of 35 participants for each group to obtain such an effect size. If the maximum dropout rate is 20% plus some residual power, we then need 45 participants for each group. After all computations, we decided that the target sample size for this study will be 90 participants.

To investigate the overall effect of the resistance exercise intervention on the change from baseline to post-intervention in the executive function composite score, a general linear mixed model (GLMM) approach with repeated measures over time (baseline and post-intervention outcomes) will be used. This statistical mixed model will include both a random intercept and slope individually for each participant and a group-by-time interaction as a fixed effect. Analyses will be primarily performed according to the intention-to-treat principle (including all participants randomized) and, in addition, based on a per-protocol principle (i.e., including only those participants with valid data for baseline and post-intervention assessments and an attendance rate to exercise program ≥ 80% of all sessions). To test the effect of the AGUEDA trial on the secondary cognitive outcomes (i.e., general cognition, verbal memory, visuospatial memory, language, and intelligence), the same analyses using an intention-to-treat principle, and GLMM analysis will be performed. All other analyses related to brain structure and function, brain amyloid load, blood biomarkers, genetics, body composition, physical function, mental health, questionnaires, among others are considered secondary or tertiary outcomes and for the mediation and moderation analysis. The plan is to test these outcomes in a similar manner as described above using an intention-to-treat principle and a GLMM approach using a group x time interaction. In addition, the mediation and moderation analysis will be performed following AGReMA (A Guideline for Reporting Mediation Analyses) recommendations (Lee et al., 2021), using the group as independent variable (wait-list vs. intervention group), executive function as outcome, and mediating and moderating variables included will be based on three levels of mechanisms proposed in the literature (i.e., molecular, brain structure and function, and behavioral levels) (Stillman et al., 2020). The potential effects of missing data will also be explored through various imputation models and sensitivity analyses. Thus, to fully appreciate the potential influence of missing data, we will perform sensitivity analyses to examine whether the imputation method changes the results. No interim analysis will be performed.

## 3. Discussion

Although exercise has the potential to improve brain health (e.g., brain structure and function) in older adults (Stillman et al., 2020; Liang et al., 2021), there is still scarce knowledge on what dose of exercise (i.e., type, duration, frequency and intensity) is sufficient for improving brain health. Well-designed intervention studies on exercise effects on brain health may also help to determine the feasibility of different types of exercise for brain guidelines, including resistance exercise. Further research in this area will help to provide rigorous, evidenced-based recommendations on how resistance exercise can facilitate improvements in brain and cognitive health.

### 3.1. Expected results

To our knowledge, only few studies has evaluated the efficacy of a resistance exercise program on brain health in cognitive healthy older adults, with a lack of including a broad understanding of several cognitive (e.g., executive function) and biological (e.g., brain Aβ) Alzheimer-related outcomes as well as its mediators/moderators. The main hypothesis is that our resistance exercise program will improve executive function, showing at least a 0.34 effect size in comparison to the wait-list control group. In addition, we also predict that molecular, brain structure and function, and behavioral changes will mediate the effect of the exercise program on executive function. Further, while there is growing evidence on the individual effects of resistance exercise on cognitive and brain health indicators, some of the expected contributions of this work to the field is the fundamental need to (i) integrate both its direct effects on cognitive indicators together with its indirect effects at the three proposed levels of mechanisms, (ii) test the interdependence of molecular-brain-behavioral mechanistic levels and, (iii) elucidate its bidirectional effects, such as feedback loops, that are likely to exist between levels.

### 3.2. Gaps and mechanisms related to the main hypothesis

Physical exercise is one of the most relevant non-pharmaceutical approaches to prevent several chronic and progressive diseases, including AD (Gleeson et al., 2011; Fiuza-Luces et al., 2013; Liang et al., 2022). While research about the effect of exercise interventions on brain health is increasing, several questions about type and dose remain inconclusive. Particularly, combined evidence on the effect of resistance exercise interventions on executive function and its mechanisms at molecular, brain structure and function and behavioral levels is largely unknown. However, isolated evidence might support the premise of our hypothesis. First, resistance exercise has shown to improve cognition, being an alternative to other types of exercises (Chang et al., 2012; Li et al., 2018; Northey et al., 2018; Landrigan et al., 2020; Gallardo-Gomez et al., 2022). Second, at a molecular level, resistance exercise is a key factor in the secretion of BDNF (Marinus et al., 2019) and IGF-1 levels in older adults, (Jiang et al., 2020) and acutely in irisin levels of young adults (Tsuchiya et al., 2015). Despite this, there is still a need for evidence on resistance exercise interventions since this type of exercise might additionally boost certain exerkines (Vints et al., 2022). Interestingly, resistance exercise in animal models triggers specific epigenomic changes of interferon pathways in the hippocampus, related to interleukin-1 beta expression, which plays a role in the inflammatory response of the brain (Urdinguio et al., 2021), and reduces neuroinflammatory cytokine expression (Kelty et al., 2022). Third, at structural and functional brain levels, only a few studies have investigated the effect of resistance exercise programs on brain structure and function in older adults. Specifically, previous evidence shows that resistance exercise influences brain volumes and morphology (Best et al., 2015; Kim et al., 2017), specifically hippocampal volumes (Broadhouse et al., 2020), white matter lesions (Bolandzadeh et al., 2015) and atrophy (Best et al., 2015), cortical thickness and functional connectivity (Suo et al., 2016), while other measures such as cerebral blood flow are understudied (Bliss et al., 2021; Smith et al., 2021). Despite this, some observational studies show that resistance exercise participation is related to cerebral blood flow in older adults (Xu et al., 2014), and there is evidence of changes in functional connectivity and cerebral blood flow in other populations (Akbar et al., 2020; Thomas et al., 2021; Huang et al., 2022). On the other hand, no previous human studies have examined the effects of resistance exercise on specific (i.e., brain Aβ) Alzheimer-related biomarkers, and few studies examined the effect on peripheral (i.e., Aβ or Tau) biomarkers (Baek et al., 2021; Hyang-Beum and Tae-Sang, 2021), with no conclusive results. Finally, at the behavioral level, resistance exercise shows effects on a broad array of mental health outcomes in older population, such as health-related quality of life (Hart and Buck, 2019), depressive (Moraes et al., 2020; Cunha et al., 2021; De Sousa et al., 2021) or anxiety symptoms (Cunha et al., 2021), and sleep (Kovacevic et al., 2018; Li et al., 2021), but the study of these measures as potential mediators of cognition are needed.

### 3.3. Limitations

Our study will provide considerable advances in the knowledge of resistance exercise effects on brain and cognitive health. However, there are some limitations that we must acknowledge. We did not perform an extensive cognitive adjudication to clinically identify cognitive status. Instead, we used STICS-m, MoCA and MMSE tests to determine cognitive status. While these are classical cognitive tests commonly used in clinical and research settings, they may have limitations in terms of sensitivity and specificity for detecting MCI. To address this limitation, we applied important education/age adjustment to secure the highest specificity in this specific population. In addition, we selected a wait-list control group, we will not be able to directly demonstrate that a resistance exercise program is more effective than other types of exercise in this population. The main goal of this RCT is to demonstrate that resistance exercise is also a type of exercise to improve cognition in older adults and to understand its underlying mechanisms. In addition, findings from the AGUEDA trial should be interpreted in the context of the sample characteristics, namely, cognitively normal older adults without physical limitation to perform exercise, and it is not possible to extrapolate to other populations.

An overarching limitation in the field is that the details of how exercise interventions are implemented are often poorly reported. Detailed prescriptions and exercise intervention characteristics are necessary to translate into clinical practice (Gallardo-Gomez et al., 2022; Hansford et al., 2022). The AGUEDA trial will overcome this limitation and will deliver a detailed and broader description of the program to generalize the prescription of resistance exercise in an older population. Importantly, one of the strengths of the AGUEDA exercise program is the minimal equipment requirement, which enables the application and translation of the results to different contexts and clinical practices. Lastly, another important characteristic of our study is the assessment of a broad spectrum of physical (e.g., fitness, body composition, and gait variability), brain (e.g., executive function, brain structure and function and mental health) and behavioral (e.g., sleep, diet, and sedentary time) indicators to fully understand mediators and moderators of resistance exercise-induced cognitive changes in older adults.

## 4. Conclusion

The AGUEDA trial will shed light on the effect of a 24-week resistance exercise intervention on executive function, and to understand its underlying mechanisms. The results of the AGUEDA trial will have important clinical implications given the increase of older adults in the population and the prevalence of cognitive decline in this age range. The AGUEDA trial will also help to establish an effective preventive strategy based on resistance exercise for delaying cognitive decline and dementia.

## Ethics statement

The trial protocol is in accordance with the principles of the Declaration of Helsinki and has been approved by the Research Ethics Board of the Andalusian Health Service (CEIM/CEI Provincial de Granada; #2317-N-19).

## Author contributions

IE-C conceived the study. IE-C, PS-U, and CM-H designed the study. PS-U, CM-H, MG-R, KE, AC, FO, and JM-G made substantial intellectual contribution to design of the study. PS-U, CM-H, CC-R, BF-G, MO-R, AC-P, and AT made substantial contributions preparation of material and protocols. PS-U, CM-H, YG-R, and ET-I substantial logistical contributions for the acquisition of data. All authors contributed intellectually and approved the final manuscript of this study protocol.

## Footnotes

1
https://github.com/aguedaprojectugr/Protocolos_AGUEDA
