# Supplementary material for: Active Gains in brain Using Exercise During Aging (AGUEDA): protocol for a randomized controlled trial
Source: Front Hum Neurosci. 2023 May 22;17:1168549. doi: 10.3389/fnhum.2023.1168549 (PMC10239947; doi:10.3389/fnhum.2023.1168549)
Supplement: Supplementary file 1 [file Table_1.DOCX]

Supplementary Material

Article Title

**P. Solis-Urra^1,2,3^, Cristina Molina-Hidalgo^4^, Yolanda García-Rivero^2,5^, Claudia Costa-Rodriguez^6^, Jose Mora-Gonzalez^1^, Beatriz Fernandez-Gamez^1^, Marcos Olvera-Rojas^1^, Andrea Coca-Pulido^1^, Angel Toval^1^, Dario Bellon^1^, Alessandro Sclafani^1^, Isabel Martín-Fuentes^1^, Eva María Triviño-Ibañez^2,5^, Carlos de Teresa^7^, Haiqing Huang^4^, George Grove^4^, Charles H. Hillman^8,9,10^, Arthur F.Kramer^8,11^, Andrés Catena^12^, Francisco B. Ortega^1,13^, Manuel Gómez-Río^2,5^, Kirk I. Erickson^4,14^,Irene Esteban-Cornejo^1^**

*** Correspondence:**Corresponding author: Patricio Solis-Urra & Irene-Esteban-Cornejo. Department of Physical Education and Sports, Faculty of Sports Science, University of Granada; Carretera de Alfacar, 21. Granada 18071, Spain; +(34) 958 24 66 51, fax: +(34) 958 24 94 28; email: [patriciosolis@ugr.es](mailto:patriciosolis@ugr.espatri) & [ireneesteban@ugr.es](mailto:ireneesteban@ugr.es)

**
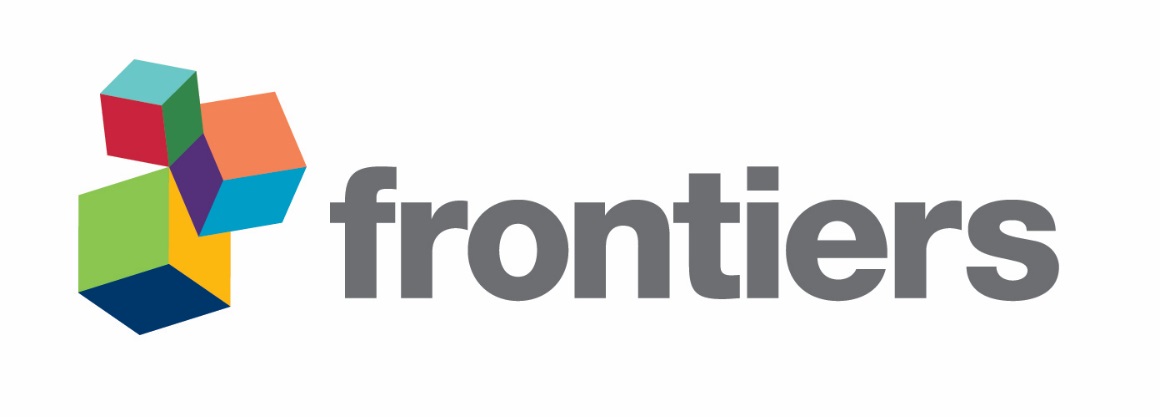
**


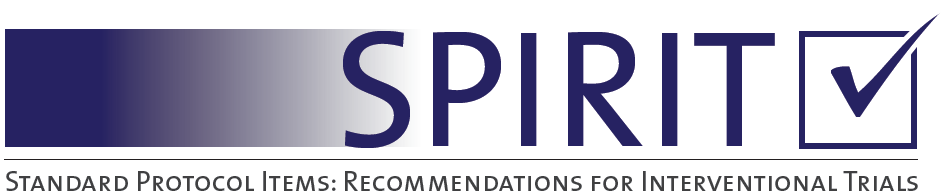
Supplementary table S1. SPIRIT Cheklist of the AGUEDA Trial.

SPIRIT 2013 Checklist: Recommended items to address in a clinical trial protocol and related documents*

| Section/item | Item No | Description | Addressed headings; pags. |
| --- | --- | --- | --- |
| **Administrative information** | | |  |
| Title | 1 | Descriptive title identifying the study design, population, interventions, and, if applicable, trial acronym | Title page; 1 |
| Trial registration | 2a | Trial identifier and registry name. If not yet registered, name of intended registry | Design and ethics; 6 |
|  | 2b | All items from the World Health Organization Trial Registration Data Set | N/A |
| Protocol version | 3 | Date and version identifier | Design and ethics; 6 |
| Funding | 4 | Sources and types of financial, material, and other support | Funding; 26 |
| Roles and responsibilities | 5a | Names, affiliations, and roles of protocol contributors | Title page/Funding; 1,26 |
|  | 5b | Name and contact information for the trial sponsor | Title page; 1 |
|  | 5c | Role of study sponsor and funders, if any, in study design; collection, management, analysis, and interpretation of data; writing of the report; and the decision to submit the report for publication, including whether they will have ultimate authority over any of these activities | Competing interest; 27 |
|  | 5d | Composition, roles, and responsibilities of the coordinating centre, steering committee, endpoint adjudication committee, data management team, and other individuals or groups overseeing the trial, if applicable (see Item 21a for data monitoring committee) | N/A |
| Introduction |  |  |  |
| Background and rationale | 6a | Description of research question and justification for undertaking the trial, including summary of relevant studies (published and unpublished) examining benefits and harms for each intervention | Introduction; 3-6 |
|  | 6b | Explanation for choice of comparators | Resistance exercise program; 17 |
| Objectives | 7 | Specific objectives or hypotheses | Introduction; 5-6 |
| Trial design | 8 | Description of trial design including type of trial (eg, parallel group, crossover, factorial, single group), allocation ratio, and framework (eg, superiority, equivalence, noninferiority, exploratory) | Design and ethics / Randomization; 6, 15 |
| Methods: Participants, interventions, and outcomes | | |  |
| Study setting | 9 | Description of study settings (eg, community clinic, academic hospital) and list of countries where data will be collected. Reference to where list of study sites can be obtained | Design and ethics; 6-7 |
| Eligibility criteria | 10 | Inclusion and exclusion criteria for participants. If applicable, eligibility criteria for study centres and individuals who will perform the interventions (eg, surgeons, psychotherapists) | Recruitment / Table 1 / Resistance exercise program; 7, 17 |
| Interventions | 11a | Interventions for each group with sufficient detail to allow replication, including how and when they will be administered | Resistance exercise program; 16-18 |
|  | 11b | Criteria for discontinuing or modifying allocated interventions for a given trial participant (eg, drug dose change in response to harms, participant request, or improving/worsening disease) | Adverse event and participation safety; 18 |
|  | 11c | Strategies to improve adherence to intervention protocols, and any procedures for monitoring adherence (eg, drug tablet return, laboratory tests) | Resistance exercise program; 17, 18 |
|  | 11d | Relevant concomitant care and interventions that are permitted or prohibited during the trial | Resistance exercise program; 18 |
| Outcomes | 12 | Primary, secondary, and other outcomes, including the specific measurement variable (eg, systolic blood pressure), analysis metric (eg, change from baseline, final value, time to event), method of aggregation (eg, median, proportion), and time point for each outcome. Explanation of the clinical relevance of chosen efficacy and harm outcomes is strongly recommended | Outcome measurements / Table 2 / Power and analytic strategy; 9, 20, 21 |
| Participant timeline | 13 | Time schedule of enrolment, interventions (including any run-ins and washouts), assessments, and visits for participants. A schematic diagram is highly recommended (see Figure) | Recruitment /  Figure 1; 7 |
| Sample size | 14 | Estimated number of participants needed to achieve study objectives and how it was determined, including clinical and statistical assumptions supporting any sample size calculations | Power and analytic strategy; 20, 21 |
| Recruitment | 15 | Strategies for achieving adequate participant enrolment to reach target sample size | Recruitment; 7 |
| **Methods: Assignment of interventions (for controlled trials)** | | |  |
| Allocation: |  |  |  |
| Sequence generation | 16a | Method of generating the allocation sequence (eg, computer-generated random numbers), and list of any factors for stratification. To reduce predictability of a random sequence, details of any planned restriction (eg, blocking) should be provided in a separate document that is unavailable to those who enrol participants or assign interventions | Randomization; 15 |
| Allocation concealment mechanism | 16b | Mechanism of implementing the allocation sequence (eg, central telephone; sequentially numbered, opaque, sealed envelopes), describing any steps to conceal the sequence until interventions are assigned | Randomization; 15 |
| Implementation | 16c | Who will generate the allocation sequence, who will enrol participants, and who will assign participants to interventions | Randomization; 15 |
| Blinding (masking) | 17a | Who will be blinded after assignment to interventions (eg, trial participants, care providers, outcome assessors, data analysts), and how | Blinding; 15, 16 |
|  | 17b | If blinded, circumstances under which unblinding is permissible, and procedure for revealing a participant’s allocated intervention during the trial | Blinding / Adverse event and participation safety; 15, 16, 18 |
| **Methods: Data collection, management, and analysis** | | |  |
| Data collection methods | 18a | Plans for assessment and collection of outcome, baseline, and other trial data, including any related processes to promote data quality (eg, duplicate measurements, training of assessors) and a description of study instruments (eg, questionnaires, laboratory tests) along with their reliability and validity, if known. Reference to where data collection forms can be found, if not in the protocol | Data management and dissemination policy / Outcome measurements; 9; 19, 20 |
|  | 18b | Plans to promote participant retention and complete follow-up, including list of any outcome data to be collected for participants who discontinue or deviate from intervention protocols | Power and analytic strategy / Adverse event and participation safety; 18, 20, 21 |
| Data management | 19 | Plans for data entry, coding, security, and storage, including any related processes to promote data quality (eg, double data entry; range checks for data values). Reference to where details of data management procedures can be found, if not in the protocol | Data management and dissemination policy; 19, 20 |
| Statistical methods | 20a | Statistical methods for analysing primary and secondary outcomes. Reference to where other details of the statistical analysis plan can be found, if not in the protocol | Power and analytic strategy; 19, 20 |
|  | 20b | Methods for any additional analyses (eg, subgroup and adjusted analyses) | Power and analytic strategy; 19, 20 |
|  | 20c | Definition of analysis population relating to protocol non-adherence (eg, as randomised analysis), and any statistical methods to handle missing data (eg, multiple imputation) | Power and analytic strategy; 19, 20 |
| **Methods: Monitoring** | | |  |
| Data monitoring | 21a | Composition of data monitoring committee (DMC); summary of its role and reporting structure; statement of whether it is independent from the sponsor and competing interests; and reference to where further details about its charter can be found, if not in the protocol. Alternatively, an explanation of why a DMC is not needed | N/A |
|  | 21b | Description of any interim analyses and stopping guidelines, including who will have access to these interim results and make the final decision to terminate the trial | Power and analytic strategy; 19, 20 |
| Harms | 22 | Plans for collecting, assessing, reporting, and managing solicited and spontaneously reported adverse events and other unintended effects of trial interventions or trial conduct | Adverse event and participation safety; 18 |
| Auditing | 23 | Frequency and procedures for auditing trial conduct, if any, and whether the process will be independent from investigators and the sponsor | N/A |
| Ethics and dissemination | | |  |
| Research ethics approval | 24 | Plans for seeking research ethics committee/institutional review board (REC/IRB) approval | Design and ethics; 6, 7 |
| Protocol amendments | 25 | Plans for communicating important protocol modifications (eg, changes to eligibility criteria, outcomes, analyses) to relevant parties (eg, investigators, REC/IRBs, trial participants, trial registries, journals, regulators) | Design and ethics; 6, 7 |
| Consent or assent | 26a | Who will obtain informed consent or assent from potential trial participants or authorised surrogates, and how (see Item 32) | Design and ethics; 6, 7 |
|  | 26b | Additional consent provisions for collection and use of participant data and biological specimens in ancillary studies, if applicable | Design and ethics / Biological samples; 6, 12 |
| Confidentiality | 27 | How personal information about potential and enrolled participants will be collected, shared, and maintained in order to protect confidentiality before, during, and after the trial | Data management and dissemination policy; 19, 20 |
| Declaration of interests | 28 | Financial and other competing interests for principal investigators for the overall trial and each study site | Competing interest; 27 |
| Access to data | 29 | Statement of who will have access to the final trial dataset, and disclosure of contractual agreements that limit such access for investigators | Data management and dissemination policy; 19, 20 |
| Ancillary and post-trial care | 30 | Provisions, if any, for ancillary and post-trial care, and for compensation to those who suffer harm from trial participation | Randomization; 15 |
| Dissemination policy | 31a | Plans for investigators and sponsor to communicate trial results to participants, healthcare professionals, the public, and other relevant groups (eg, via publication, reporting in results databases, or other data sharing arrangements), including any publication restrictions | Data management and dissemination policy; 19, 20 |
|  | 31b | Authorship eligibility guidelines and any intended use of professional writers | Data management and dissemination policy; 19, 20 |
|  | 31c | Plans, if any, for granting public access to the full protocol, participant-level dataset, and statistical code | Data management and dissemination policy; 19, 20 |
| Appendices |  |  |  |
| Informed consent materials | 32 | Model consent form and other related documentation given to participants and authorised surrogates | Design and ethics; 6, 7 |
| Biological specimens | 33 | Plans for collection, laboratory evaluation, and storage of biological specimens for genetic or molecular analysis in the current trial and for future use in ancillary studies, if applicable | Design and ethics / Biological samples; 6, 7, 11, 12 |

*It is strongly recommended that this checklist be read in conjunction with the SPIRIT 2013 Explanation & Elaboration for important clarification on the items. Amendments to the protocol should be tracked and dated. The SPIRIT checklist is copyrighted by the SPIRIT Group under the Creative Commons “[Attribution-NonCommercial-NoDerivs 3.0 Unported](http://www.creativecommons.org/licenses/by-nc-nd/3.0/)” license.
